# Closing the Gap Between Evidence and Action: Lessons From a Dramatic Increase in Access to Alcohol

**DOI:** 10.1111/dar.70170

**Published:** 2026-05-14

**Authors:** Norman Giesbrecht, Elizabeth K. Farkouh

**Affiliations:** ^1^ Centre for Addiction and Mental Health Institute for Mental Health Policy Research Toronto Ontario Canada; ^2^ Dalla Lana School of Public Health University of Toronto Toronto Ontario Canada; ^3^ Brigham & Women's Hospital Harvard Medical School Boston Massachusetts USA; ^4^ Canadian Institute for Substance Use Research Victoria British Columbia Canada

**Keywords:** alcohol availability, alcohol policy, Canada, mitigation

## Abstract

Alcohol is a leading cause of death, disability, disease, trauma and social problems. In Canada, alcohol consumption is associated with 17,000 deaths annually, with costs from alcohol exceeding revenues in each jurisdiction. Leading health organisations point to policies shown to be effective in reducing alcohol harms. Yet there is a substantial and growing gap between evidence of effective policies to reduce alcohol harms and actual policies being implemented in Canada and elsewhere. This commentary critically examines one such example: the Ontario government's 2023 decision to significantly expand off‐premise alcohol sales, involving up to 8500 new off‐premise outlets (+290%). There were several ways in which this decision neglected public health. First, both international and Canadian‐based research demonstrates that greater physical access to alcohol is associated with increased chronic disease, acute harms and deaths. However, this publicly available evidence did not appear to inform the Ontario provincial government's plans to dramatically increase alcohol availability, and the government did not appear to conduct a priori estimates of health and social impacts. Second, despite concerns from leading public health associations, the plan was not modified. Finally, the plan proceeded with minimal mitigation interventions and without an evaluation protocol. This illustrative case of evidence‐neglect in public health policy can inform policymaking in other jurisdictions. Governments considering changes to alcohol policies should utilise available evidence to conduct an a priori assessment of potential risks and benefits, consult with relevant public health stakeholders, enact a robust, multi‐dimensional mitigation plan, and implement an objective and comprehensive evaluation protocol.

## 1 Introduction

Alcohol consumption is a major contributor to deaths, disability, and social problems worldwide [1]. In Canada, over 17,000 deaths were attributable to alcohol use in 2020, and recent evidence demonstrates that since the onset of the COVID‐19 pandemic, alcohol‐attributable mortality has increased by 18% [2, 3].

Alcohol control policies have demonstrated benefit in preventing alcohol‐related harm [4, 5, 6]; however, in Canada, uptake of evidence‐based alcohol policies remains poor, both at the federal and provincial/territorial level [7, 8, 9, 10]. In fact, in many cases, political decisions reverse course, such as increasing physical access, neglecting to implement taxes on alcohol that keep pace with inflation, and failing to implement strong controls on alcohol marketing [7].

A dynamic underlying these decisions is the contrast between a commercial (or populist) orientation to alcohol policy planning and implementation versus one informed by an interest in protecting health and promoting safety. The former places high value on convenience to the consumer and the agenda of retailers and producers, while the latter values evidence of potential impacts on the total population and vulnerable sectors, as well as attendant costs [5].

In December 2023, the Province of Ontario Premier, Doug Ford, announced plans to dramatically increase off‐premise retail outlets permitted to sell alcohol [11]. The rationale for this decision was multi‐faceted and included delivering on an election promise, increasing consumer choice and convenience, supporting convenience store owners, increasing shelf space for craft brewers, and allowing for more competitive retail pricing [12]. The policy change was also part of a broader wave of alcohol deregulation across Canadian provinces and territories following the COVID‐19 pandemic [9].

Initially, this change was planned to begin in January 2026, when an agreement between the government and a large brewers' network that held an alcohol monopoly would expire [13]. However, in May 2024 the Premier announced that the expansion would be accelerated. Specifically, up to 8500 new outlets—including corner or convenience stores and big box stores—could sell beer, wine, cider and ready‐to‐drink alcoholic beverages by Fall 2024 [12]. This change in timeline involved a requirement to pay a $225 million penalty to the brewers' network [13]. A further analysis estimates that the decision to fast‐track the alcohol expansion cost the province over $600 M [13]. A subsequent analysis by the Financial Accountability Office of Ontario projected a net cost of $1.4 billion between 2024 and 2030 [14].

The policy change not only increased the number of alcohol outlets but involved alcohol sales in new types of outlets. Prior to the expansion, off‐premise alcohol sales were allowed in beer store network stores, Ontario wine store outlets, the Liquor Control Board of Ontario (LCBO) outlets and, since 2015, supermarkets. With the expansion, convenience stores and big‐box stores were added, as well as additional supermarkets. Before the change, LCBO outlets were the only off‐premise system that sold spirits. However, with the expansion, new private outlets were permitted to sell ready‐to‐drink cocktails, coolers and hard seltzers [15].

As of October 2024, there was already a significant surge in the number of outlets, with 4700 convenience stores and 850 grocery stores licensed to sell alcohol, in addition to pre‐existing LCBO, Beer Store and wine store outlets, compared to 2935 total outlets prior to the expansion [16]. The expansion could eventually result in up to a 290% increase in off‐premise retail density (8500 new outlets) [17], the largest increase in physical access to alcohol in any Canadian jurisdiction since the end of prohibition [18].

This commentary examines this policy decision and whether the government acknowledged the international and Canadian evidence, whether they conducted an a priori health and social impact assessment, whether they adjusted their plans based on the advice of public health experts, which effective mitigation strategies were implemented, and whether a robust wide‐ranging evaluation plan was put in place.

## 2 Evidence of Physical Availability on Alcohol Consumption or Harm

There is substantial international research noting that alcohol access, including the density of alcohol outlets in a given area, is significantly and positively associated with alcohol consumption/sales [5, 19, 20, 21]. By extension, there are also consistent associations between greater alcohol access (e.g., outlet density, allowance of alcohol in convenience and grocery stores) and various alcohol harms [5, 21, 22] including alcohol‐impaired driving [20]. In “Alcohol and Public Policy: No Ordinary Commodity” an international group of alcohol policy experts concluded that “studies of natural experiments, especially of large‐scale changes in alcohol availability show relatively consistent impact on consumption and harm rates” ([5], 135).

Like the international evidence, longitudinal data from Canadian jurisdictions also demonstrates that increased alcohol access and privatisation is associated with an increase in consumption and harm. For example, when Alberta underwent staged privatisation of alcohol sales from 1985 to 1994, there was a significant increase in suicide deaths [23]. In 2002, British Columbia experienced a 33% increase in privately owned liquor stores, which was associated with an increase in alcohol consumption [24] and harm, including a rise in alcohol‐attributable hospital admissions [25], alcohol‐related deaths [26, 27]; and alcohol‐involved traffic violations [28]. In Ontario, Myran et al. found that the allowance of alcohol sales in grocery stores in late 2015 was associated with an increase in the number of alcohol‐attributable emergency department visits [29]. Likewise, Gohar et al. [30] found that the risk of an Ontario secondary student transitioning from alcohol abstinence to high‐risk alcohol use was 1.7 times greater among students that were exposed to grocery stores allowed to sell alcohol versus those that were not.

Despite the available data (as of 2023) from Canada, and the global context demonstrating the risks of alcohol expansion, there is no public information to suggest that the Ontario government's policymakers formally considered this evidence in their decision to expand alcohol sales. In fact, to our knowledge, the Ontario government undertook no formal public consultation process. There is also no evidence that they conducted a priori assessment of health and social impacts.

## 3 Responses by Public Health Leadership

In March 2024, the Ontario Chief Medical Officer of Health's (MOH) report recommended that Ontario “establish and maintain a moratorium on alcohol privatization;” “implement an evidence‐informed, quantity based system to manage outlet density;” and “maintain or reduce current levels of outlet density.” [31]. The premier publicly and summarily rejected his Chief MOH's advice and implied that the expansion would proceed as originally planned [32].

Additional concerns raised by research organisations [33, 34, 35], in op‐ed articles [36], through letters to the editor [37, 38] and in commentaries [16, 39] did not lead to substantial changes.

There are several potential reasons why the government ignored public health warnings. First, the government appears to have prioritised consumer access, promoting the idea that the expansion promoted “choice and convenience” [12]. The government also falsely implied that the changes would put Ontario in line with the level of access available in all other Canadian and international jurisdictions [40]. While there are no publicly available data on lobbying activities, it is also possible that alcohol industry lobbying played a role.

## 4 Prevention and Mitigation

As part of the alcohol expansion, the government pledged $10 M dollars over a 5‐year period “to support social responsibility and public health efforts to ensure alcohol continues to be sold and consumed safely in the expanded marketplace” [12]. However, there was no public information regarding the specific interventions included in this promise, and the pledge works out to $2 million per year. With over 5000 new outlets as of October 2024, the pledge results in less than $400 spent on social responsibility/public health initiatives per new outlet per year.

Notably, as was the case prior to the policy change, all persons selling alcohol will be required to take the standard server training course [12]. However, the Government of Ontario and its agencies failed to implement several other important prevention or mitigation strategies. To our knowledge, no new policies were implemented that were designed to curtail the projected harm from a dramatic increase in access to alcohol. For example, there were no plans to substantially increase the number of inspectors, in response to a projected 290% increase in off‐premise outlets, to reduce sales to minors or intoxicated patrons. Table 1 provides an overview of other potential strategies that could have been (but were not) implemented to mitigate harms from the alcohol sales expansion. To offset such a large increase in access to alcohol, several high‐impact interventions would be needed, such as increased alcohol taxation, minimum pricing increases, and reduction in hours of sale, among others.

**TABLE 1:** Potential mitigation strategies to prevent harms of increasing access to alcohol in Ontario.

Domain	Potential mitigation strategies	Potential impact^a^
Rationale and planning	Consult with public health groups before developing a plan. Work with Brewers and Liquor Board to facilitate better access to shelf space for craft brewers (as opposed to expanding outlets). Assess research on experiences elsewhere; avoid relying on anecdotes. Conduct full cost and social impact accounting (e.g., modelling study, a priori) before finalising plan.	Medium
Implementation	Have a higher licensing fee to help offset expected costs associated with crime and health impacts. Have licensing fees that are scaled to the expected shelf space per outlet (e.g., higher fees for big box outlets or large grocery stores).	High
Timeline	Delay the implementation until January 2026 and save taxpayers $225 million [41]. Have a phased in model with an annual limit of new outlets, with ongoing assessment of risks, harms and costs [5].	High
Municipalities	Give municipalities the option to opt out of the plan and set limits on the number of total retail outlets per capita per municipality [42].	High
Location	Set limits on how close retail outlets can be to schools, clinics, hospitals, day care centres and substance use treatment centres [42].	Low
Server training	Enhance server training because private retail outlets have been shown to not be as vigilant about selling alcohol to underage persons or intoxicated patrons [43].	Medium
Raising consumer awareness	Mandate enhanced warning labels on alcohol containers so that potential consumers are better aware of the hazards and risks [42]. Mandate warning signs in all retail outlets selling alcohol [8, 42].	Medium
Hours of sale	Reduce hours of sale for off‐premise outlets [8, 42].	High
Pricing	Maintain minimum pricing for alcohol and ensure minimum prices are indexed to inflation [8, 42].	High
Provincial alcohol strategy	Have a provincial alcohol strategy that examines all proposals carefully and thoughtfully prior to implementation [8, 42].	Medium

^a^ Estimated potential impact based on Babor et al. [5], Farkouh et al. [7] and Priore et al. [9].

## 5 Evaluation of Impacts

To date, there have been no government announcements of an evaluation plan. A comprehensive plan, using survey data, official statistics on alcohol sales by outlet, health and law enforcement impacts, and key informant interviews, should include an analysis of the following: whether the policy change achieved its goals, including economic ones, impacts on health and safety, and whether the pledged $10 million mitigated harms.

## 6 Conclusion

In conclusion, it appears that in expanding off‐premise alcohol outlets, the Ontario provincial government did not draw on the international or Canadian evidence demonstrating the risks associated with an increase in physical access to alcohol. It apparently did not conduct an a priori health and social impact assessment, and, in contrast to other international jurisdictions which have conducted public consultation on alcohol policy changes, no such process was done for this large‐scale policy change in Ontario [44, 45]. Concerns raised by public health organisations and the Ontario MOH were either ignored or dismissed, and several potential mitigation or prevention strategies have not, to date, been introduced.

From a public health and safety perspective, the alcohol expansion plan and its components are deeply flawed. Many questions remain unanswered: will these changes lead to more harms, and if so, to what extent? What will be the impact on specific harms like emergency room visits, underage consumption, alcohol use disorder, chronic disease, intimate partner violence and impaired driving incidents, among many others? Other potential downstream impacts of this policy change, such as further normalisation of alcohol use and increased burden on the healthcare system, also remain to be seen. A comprehensive evaluation plan would be helpful in assessing these impacts.

To reduce harm from increased access to alcohol, policy‐planning should evaluate the totality of evidence from the most relevant jurisdictions and conduct an a priori assessment of potential benefits and risks, as well as which communities are likely to be most impacted. Public health stakeholders should have a seat at the table when alcohol policy changes are being proposed, debated, and decided upon. Additionally, a detailed, multi‐dimensional, and appropriately funded mitigation plan should be in place before a change in policy is enacted. Finally, any major changes in alcohol policy should not proceed without a high‐quality, robust evaluation plan. This case of massive alcohol expansion in Canada's most populous province has implications for alcohol harm prevention planning in other Canadian and international jurisdictions.

## Author Contributions

Both authors (N.G., E.K.F.) contributed to conceptualisation, writing – original draft, writing – review and editing, and project administration.

## Funding

The authors have nothing to report.

## Conflicts of Interest

The authors declare no conflicts of interest.

## Data Availability

Data sharing not applicable to this article as no datasets were generated or analysed during the current study.

## References

[1] World Health Organization , “Alcohol,” 2024, https://www.who.int/news‐room/fact‐sheets/detail/alcohol.

[2] Canadian Substance Use Costs and Harms Working Group , “Canadian Substance Use Costs and Harms 2007–2020,” 2023, https://csuch.ca/resources/costs‐and‐harms/.

[3] Y. Shi , K. Macrae , M. de Groh , W. Thompson , and T. Stockwell , “Mortality and Hospitalizations Fully Attributable to Alcohol Use Before Versus During the COVID‐19 Pandemic in Canada,” CMAJ 197, no. 4 (2025): E87–e95, 10.1503/cmaj.241146. 39900361 PMC11790301

[4] P. Anderson , D. Chisholm , and D. C. Fuhr , “Effectiveness and Cost‐Effectiveness of Policies and Programmes to Reduce the Harm Caused by Alcohol,” Lancet 373, no. 9682 (2009): 2234–2246, 10.1016/s0140-6736(09)60744-3. 19560605

[5] T. F. Babor , S. Casswell , K. Graham , et al., Alcohol: No Ordinary Commodity: Research and Public Policy, 3rd ed. (Oxford University Press, 2022).

[6] D. Chisholm , D. Moro , M. Bertram , et al., “Are the “Best Buys” for Alcohol Control Still Valid? An Update on the Comparative Cost‐Effectiveness of Alcohol Control Strategies at the Global Level,” Journal of Studies on Alcohol and Drugs 79, no. 4 (2018): 514–522. 30079865

[7] E. K. Farkouh , K. Vallance , A. Wettlaufer , et al., “An Assessment of Federal Alcohol Policies in Canada and Priority Recommendations: Results From the 3rd Canadian Alcohol Policy Evaluation Project,” Canadian Journal of Public Health 115, no. 4 (2024): 640–653, 10.17269/s41997-024-00889-3. 38739320 PMC11303602

[8] T. Naimi , T. Stockwell , N. Giesbrecht , et al., “Canadian Alcohol Policy Evaluation (CAPE) 3.0 Project: Policy Domain Results Summary (Provincial/Territorial),” 2023.

[9] I. Priore , N. Vishnevsky , E. K. Farkouh , et al., “Provincial and Territorial Results and Recommendations From the Canadian Alcohol Policy Evaluation Project: Room for Improvement,” Canadian Journal of Public Health 117 (2025): 113–124, 10.17269/s41997-025-01061-1. 40506630 PMC13001149

[10] K. Vallance , T. Stockwell , A. Wettlaufer , et al., “The Canadian Alcohol Policy Evaluation Project: Findings From a Review of Provincial and Territorial Alcohol Policies,” Drug and Alcohol Review 40, no. 6 (2021): 937–945, 10.1111/dar.13251. 33543532

[11] M. Crawley , “Billions at Stake as Doug Ford Government Prepares to Change Booze Retailing in Ontario,” 2023, https://www.cbc.ca/news/canada/toronto/ontario‐beer‐wine‐retail‐lcbo‐doug‐ford‐convenience‐store‐1.7035550.

[12] Office of the Premier , “Ontario Delivering Choice and Convenience by Expanding the Sale of Alcoholic Beverages Starting This Summer,” 2024, https://news.ontario.ca/en/release/1004633/ontario‐delivering‐choice‐and‐convenience‐by‐expanding‐the‐sale‐of‐alcoholic‐beverages‐starting‐this‐summer.

[13] CBC , “Ford's Push to Expand Alcohol Sales Early to Cost Province $612 M, Rof Budget Watchdog Says,” 2025, https://www.cbc.ca/news/canada/toronto/ford‐alcohol‐beer‐wine‐booze‐cost‐taxpayers‐1.7442250.

[14] Financial Accountability Office of Ontario , “The Financial Impact of Expanding the Beverage Alcohol Marketplace in Ontario [Review of the Financial Impact of Expanding the Beverage Alcohol Marketplace in Ontario],” 2025, https://fao‐on.org/en/report/alcohol‐sales‐expansion/.

[15] Ontario Ministry of Finance , “Where to Buy Alcoholic Beverages,” 2024, https://www.ontario.ca/page/where‐buy‐alcoholic‐beverages.

[16] N. Giesbrecht and D. T. Myran , “Harms and Costs of Proposed Changes in How Alcohol is Sold in Ontario,” CMAJ 196, no. 13 (2024): e447–e448, 10.1503/cmaj.240069. 38589028 PMC11001388

[17] Alcohol & Health , “Infographic: The Ontario Government is Planning a Nearly 300% Increase in Alcohol Retail Locations,” https://alcoholandhealth.ca/wp‐content/uploads/2024/08/Alcohol‐retail‐infographic_Final.pdf.

[18] Office of the Premier , “All Ontario Grocery and Big‐Box Stores Now Able to Sell Alcoholic Beverages,” 2024, https://news.ontario.ca/en/release/1005247/all‐ontario‐grocery‐and‐big‐box‐stores‐now‐able‐to‐sell‐alcoholic‐beverages.

[19] C. A. Campbell , R. A. Hahn , R. Elder , et al., “The Effectiveness of Limiting Alcohol Outlet Density as a Means of Reducing Excessive Alcohol Consumption and Alcohol‐Related Harms,” American Journal of Preventive Medicine 37, no. 6 (2009): 556–569, 10.1016/j.amepre.2009.09.028. 19944925

[20] G. Edwards , P. Anderson , T. F. Babor , et al., Alcohol Policy and the Public Good (Oxford University Press, 1994), https://books.google.com/books?id=235HAAAAMAAJ.

[21] S. Popova , N. Giesbrecht , D. Bekmuradov , and J. Patra , “Hours and Days of Sale and Density of Alcohol Outlets: Impacts on Alcohol Consumption and Damage: A Systematic Review,” Alcohol and Alcoholism (Oxford, Oxfordshire) 44, no. 5 (2009): 500–516, 10.1093/alcalc/agp054. 19734159

[22] D. Fone , J. Morgan , R. Fry , et al., “Change in Alcohol Outlet Density and Alcohol‐Related Harm to Population Health (CHALICE): A Compreehensive Record‐Linked Database Study in Wales,” Public Health Research 4 (2016): 1–184, 10.3310/phr04030. 27054222

[23] R. F. Zalcman and R. E. Mann , “The Effects of Privatization of Alcohol Sales in Alberta on Suicide Mortality Rates,” Contemporary Drug Problems 34, no. 4 (2007): 589–609, 10.1177/009145090703400405.

[24] T. Stockwell , J. Zhao , S. Macdonald , B. Pakula , P. Gruenewald , and H. Holdder , “Changes in Per Capita Alcohol Sales During the Partial Privatization of British Columbia's Retail Alcohol Monopoly 2003–2008: A Muli‐Level Area Analysis,” Addiction 104, no. 1 (2009): 1827–1836. 19681801 10.1111/j.1360-0443.2009.02658.x

[25] T. Stockwell , J. Zhao , G. Martin , et al., “Minimum Alcohol Prices and Outlet Densities in British Columbia, Canada: Estimated Impacts on Alcohol‐Attributable Hospital Admissions,” American Journal of Public Health 103, no. 11 (2013): 2014–2020, 10.2105/ajph.2013.301289. 23597383 PMC3828694

[26] T. Stockwell , J. Zhao , S. Macdonald , et al., “Impact on Alcohol‐Related Mortality of a Rapid Rise in the Density of Private Liquor Outlets in British Columbia: A Local Area Multi‐Level Analysis,” Addiction 106, no. 4 (2011): 768–776, 10.1111/j.1360-0443.2010.03331.x. 21244541

[27] J. Zhao , T. Stockwell , G. Martin , et al., “The Relationship Between Minimum Alcohol Prices, Outlet Densities and Alcohol‐Attributable Deaths in British Columbia, 2002–09,” Addiction 108, no. 6 (2013): 1059–1069, 10.1111/add.12139. 23398533

[28] R. Stockwell , J. Zhao , M. M. Gruenewald PJ , M. S. Ponicki WR , and G. Martin , “Relationships Between Minimum Alcohol Pricing and Crime During Partial Privatization of a Canadian Government Alcohol Monopoly,” Journal of Studies on Alcohol 76, no. 4 (2015): 68–634. 10.15288/jsad.2015.76.628 PMC4495080 26098040

[29] D. T. Myran , J. T. Chen , N. Giesbrecht , and V. W. Rees , “The Association Between Alcohol Access and Alcohol‐Attributable Emergency Department Visits in Ontario, Canada,” Addiction 114, no. 7 (2019): 1183–1191, 10.1111/add.14597. 30924983

[30] M. R. Gohar , R. J. Cook , J. A. Dubin , and S. T. Leatherdale , “The Impact of an Alcohol Policy Change on on Developmental Trjactories of Youth Alcohol Use: Examination of a Experiment in Canada,” Canadian Journal of Public Health 112, no. 2 (2021): 210218. 10.17269/s41997-020-00366-7 PMC7910382 32761543

[31] Balancing Act , “An All‐of‐Society Approach to Substance Use and Harms,” Ontario, 2024, https://www.ontario.ca/files/2024‐04/moh‐cmoh‐annual‐report‐2023‐en‐2024‐04‐02.pdf.

[32] M. Talbot , “‘We Disagree’: Ford Clashes With Medical Officer on Decriminalizing Hard Drugs, Raising Drinking Age,” 2024, https://toronto.citynews.ca/2024/04/03/ford‐medical‐officer‐decriminalizing‐drugs‐raising‐drinking‐age/.

[33] Centre for Addiction and Mental Health , “CAMH Statement About Alcohol Retail Expansion in Ontario,” 2023, https://www.camh.ca/en/camh‐news‐and‐stories/camh‐statement‐about‐alcohol‐retail‐expansion‐in‐ontario.

[34] CMHA , “Coalition Urges Immediate Action on Health Care Strategy Following Alcohol Retail Expansion,” 2024, https://ontario.cmha.ca/news/coalition‐urges‐immediate‐action‐on‐health‐care‐strategy‐following‐alcohol‐retail‐expansio/.

[35] F. Kutty , “Doug Ford's False Promise of Convenience in Reality Promotes Dangerous Alcohol Consumption,” Toronto Star, 2025, https://www.thestar.com/opinion/contributors/doug‐ford‐s‐false‐promise‐of‐convenience‐in‐reality‐promotes‐dangerous‐alcohol‐consumption/article_35fed570‐7ab5‐11ef‐8c8e‐6bc4c2ee78e6.html.

[36] I. Gorfinkel , “Convenient Access to Alcohol Is Going to Cost Us,” TVO Today, 2024, https://www.tvo.org/article/convenient‐access‐to‐alcohol‐is‐going‐to‐cost‐us.

[37] J. Broadbent , “LETTER: Ford's Effort to Gut LCBO Will Do Nothing to Help Consumers,” 2024, https://www.sootoday.com/letters‐to‐the‐editor/letter‐fords‐effort‐to‐gut‐lcbo‐will‐do‐nothing‐to‐help‐consumers‐9212196.

[38] The Globe and Mail , “$225‐Million to Cancel Ontario's Beer Store Contract, Plus Other Letters for June 2: ‘I Am a Responsible Drinker (I Think) and I Can Wait a Year So We Can Save That Money’,” The Globe and Mail, 2024, https://www.theglobeandmail.com/opinion/letters/article‐ontario‐beer‐store‐break‐contract/?login=true.

[39] R. Robinson , “Do We Really Want Ontarians to Drink More? Canadian Centre for Policy Alternatives,” 2024, https://www.policyalternatives.ca/news‐research/do‐we‐really‐want‐ontarians‐to‐drink‐more/.

[40] The Canadian Press , “Ontario Speeds Up Alcohol Expansion,” 2024, https://www.tvo.org/article/ontario‐speeds‐up‐alcohol‐expansion.

[41] J. Gray , “Ontario to Pay $225‐Million to Beer Store to Speed Up Corner‐Store Sales,” Globe and Mail, May 24, 2024, https://www.theglobeandmail.com/canada/article‐ontario‐beer‐corner‐stores‐september/.

[42] E. Farkouh , T. Price , K. Vallance , et al., “Canadian Alcohol Policy Evaluation (CAPE) 3.0: Methodology and Evidence (Federal and Provincial/Territorial),” 2023, https://www.uvic.ca/research/centres/cisur/assets/docs/cape/cape3/methodology‐en.pdf.

[43] W. C. Kerr and B. L. Barnett , “Alcohol Retailing Systems: Private vs. Government Control,” in Prevention of Alcohol‐Related Problems: Evidence and Community‐Based Initiatives, ed. N. Giesbrecht and L. M. Bosma (APHA Press, 2018), 137–150.

[44] Alcohol , “Minimum Unit Pricing (MUP): Continuation and Future Pricing: Consultation,” n.d, https://consult.gov.scot/population‐health/review‐of‐the‐minimum‐unit‐pricing‐and‐contination/.

[45] A. Baxter , “Government Consultations,” House of Commons Library, 2025, https://commonslibrary.parliament.uk/research‐briefings/cbp‐10190/.

